# Polymer Engineering Enables High Linear Capacity Fiber Electrodes by Microenvironment Regulation

**DOI:** 10.1002/advs.202309461

**Published:** 2024-04-26

**Authors:** Yuan Li, Yibo Wang, Yan Liu, Fang Yan, Zhenwei Zhu, Xibang Chen, Jingyi Qiu, Hao Zhang, Gaoping Cao

**Affiliations:** ^1^ Research Institute of Chemical Defense Beijing 100191 China; ^2^ School of Chemistry and Biological Engineering Institute for Advanced Materials and Technology University of Science and Technology Beijing Beijing 100083 China

**Keywords:** flexible fiber electrodes, high linear capacity, ionic dynamics, microenvironment, polymer engineering

## Abstract

Unlike bulky and rigid traditional power systems, 1D fiber batteries possess appealing features such as flexibility and adaptability, which are promising for use in wearable electronic devices. However, the performance and energy density fiber batteries are limited by the contradiction between ionic transfer and robust structure of fiber electrodes. Herein, these problems are addressed via polymer engineering to regulate the microenvironment in electrodes, realizing high‐linear‐capacity thick fiber electrodes with excellent cycling performance. The porosity of the electrodes is regulated using polymer crosslink networks designed with various components, and lithium‐ion transfer is optimized through ether‐abundant polymer chains. Furthermore, reinforced covalent bonding with carbon nanotube networks is established based on the modified functional groups of polymer networks. The multiscale optimizations of the porous structure, ionic transportation, and covalent bonding network enhance the lithium‐ion dynamics property and structural stability. Therefore, ultrahigh linear‐capacity fiber electrodes (17.8 mAh m^−1^) can be fabricated on a large scale and exhibit excellent stability (92.8% after 800 cycles), demonstrating obvious superiority among the reported fiber electrodes. Moreover, this study highlights the high effectiveness of polymer regulation in fiber electrodes and offers new avenues for designing next‐generation wearable energy‐storage systems.

## Graphical abstract

A hybrid polymer network is designed to optimize the ionic dynamics via porosity management and ether‐abundant polymer chains. Meanwhile, the covalent bonding of the polymer networks with CNTs also achieving robust structure. The balance between ionic dynamic and mechanical stability largely promotes the linear capacity and performance of the fiber electrodes to offer long‐term power for wearable devices.

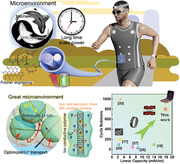

## 1 Introduction

Due to informatization and intelligence, reliance on portable and wearable electronic devices has driven urgent demands for powerful, flexible, and compact energy solutions.^[^ ^1^, ^2^ ^]^ Lithium‐ion batteries (LIBs) are widely used daily because of their mature production technology, no memory effect, and high energy density. However, traditional planar Li‐ion batteries, which have bulky and rigid structures, cannot be integrated into flexible wearable systems.^[^ ^3^, ^4^, ^5^, ^6^ ^]^ Fiber batteries with a 1D architecture have emerged as promising candidates for wearable devices’ energy systems to realize easy integration, miniaturization, softness, and breathability.^[^ ^7^, ^8^, ^9^, ^10^ ^]^ Thus, as an essential component in fiber batteries, the long‐term power requirements of wearable devices ask for exploiting fabrication techniques and materials for fiber electrodes, pursuing higher energy density, and cycling life.^[^ ^11^, ^12^ ^]^ Although various active materials and loading methods have been applied to fabricate electrodes, the energy density is still far from demand, especially for future wearable miniature communication and display devices with multifunction integration.^[^ ^13^ ^]^

Among various fabrication methods for fiber electrodes, the solution‐extrusion method has attracted attention owing to its convenient and large‐scale production ability.^[^ ^14^, ^15^ ^]^ Conductive materials and polymers construct the fiber architecture and load active material inside fibers with tunable active material ratios and designable microstructures. This offers the opportunity to obtain fiber electrodes with higher mass loading.^[^ ^16^, ^17^ ^]^ Several works have increased the active material ratio of fiber electrodes to enhance energy density. For example, Ling et al. reported an ingenious functional ink for fabricating high‐mass‐loading electrodes.^[^ ^18^ ^]^ Pyrrole‐modified reduced graphene oxide was induced to prevent the agglomeration of carbon nanotubes (CNTs), improving the content of the active material to 75%. In our previous work, we also achieved a high active material ratio by displacing the polymer binder to in situ interface binding.^[^ ^19^ ^]^ However, the linear capacity of electrodes is still severely limited by the diameter (thickness) of fiber electrodes^[^ ^20^, ^21^, ^22^ ^]^ and needs to be enhanced through innovative material and structure design.

To date, few works could achieve thick fiber electrodes, primarily due to the poor ionic dynamics compared with planar architecture. As ion transfer directed to the center would be hindered by reduced graphene oxide (rGO) or MXene in the dense structure,^[^ ^22^, ^23^, ^24^, ^25^ ^]^ polymers might play a crucial role in proving ion transport channels in fiber electrodes. However, achieving rheotropic adjustment and durability under deformation requires a significant amount of polymer to build a durable structure,^[^ ^26^, ^27^ ^]^ thereby limiting the application of fragile aerogel structures with excessively high porosity.^[^ ^28^ ^]^ In this context, the commonly used polyvinylidene difluoride (PVDF) binder fails to meet the demands of ionic dynamics with the fiber electrode diameter (thickness) increases.^[^ ^29^ ^]^ Therefore, the design of polymer cross‐link networks in fiber electrodes should be tailored to enhance ionic transportation without sacrificing structural durability. However, this aspect has not been thoroughly explored by researchers and could yield significant advancements for high‐energy‐density fibrous LIBs using the solution‐extrusion method.

**Figure 1:**
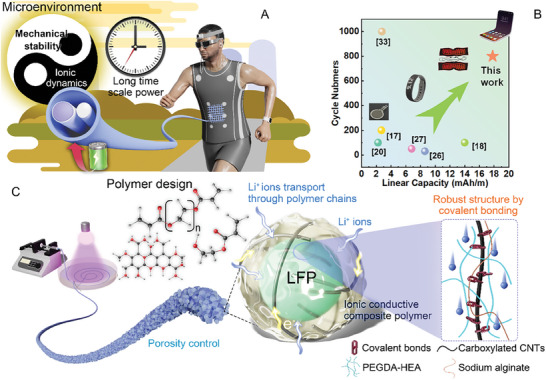
Schematic of microenvironment management via polymer engineering to enhance the property of fiber electrodes. A) Balance of ionic dynamics and robust structure in the microenvironment of fiber electrodes endow fiber batteries with higher energy and cycling life to serve as a long‐term power for wearable electronic devices. B) Linear capacity and cycling life superiority of prepared fiber electrodes.^[^ ^17^, ^18^, ^20^, ^26^, ^27^, ^33^ ^]^ C) Regulation of the microenvironment, including porosity, ion transport, and covalent bonding, in fiber electrodes by polymer design.

In this study, the crucial roles of polymer crosslinking networks in fiber electrodes were highlighted. An innovative polymer engineering strategy was exploited to regulate microenvironments^[^ ^30^ ^]^ (realizing high ionic dynamics and robust structure) to successfully achieve high linear capacity and excellent cycling life (**Figure** **1**). As a favored ionic conductive polymer material,^[^ ^31^ ^]^ poly (ethylene glycol) diacrylate (PEGDA) own abundant ether bonds to transport Li^+^ via association/disassociation and polymer chain movement. It was first applied in fiber electrode fabrication as the predominant constituent of polymer to optimize ionic transport ability inside fiber electrodes. To overcome the inhibition of PEGDA polymerization due to vast CNTs and LiFePO_4_ (LFP), sodium alginate (SA) and 2‐hydroxyethyl acrylate (HEA) were introduced in PEGDA solution (named PSH polymer), realizing quick architecture shaping and constructing robust hybrid networks. During the process, the initial fast electrostatic interaction‐driven crosslinks of SA ensure the rapid and continuous prototyping of fiber electrodes, following the time‐consuming polymerization process of PEGDA.^[^ ^32^ ^]^ Additionally, SA and HEA adjust the rheological properties of the inks and decrease the degree of crosslinking and crystallinity, thereby enhancing porosity and ion transport ability. Moreover, the HEA‐functioned PEGDA could establish covalent bonding with carboxylated CNTs, resulting in stable co‐continue ion and electron transfer channels. Therefore, the optimized ionic dynamic enabled flexible thick fiber electrodes with a linear capacity of 17.8 mAh m^−1^, and covalent networks promise high stability of the electrodes (92.8% capacity retention after 800 cycles) with less inner stress due to decreased polarization. Compared with reported works, this superiority endows it with more application potential as long‐term power for wearable devices.^[^ ^17^, ^18^, ^20^, ^26^, ^27^, ^33^ ^]^ We envision that the proposed strategy for the scalable fabrication of high‐energy‐density fibrous LIBs via polymer engineering would offer a new route to develop energy fabric for emerging wearable device demands.

## 2 Results and Discussion

### 2.1 Rheological Properties and Fabrication Processes

**Figure 2:**
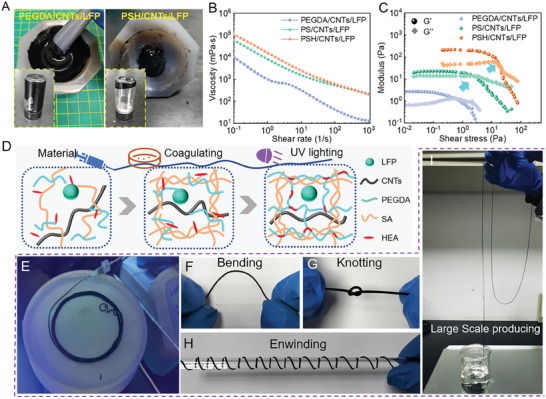
Rheological properties of inks and fabrication processes of fiber electrodes. A) Photograph of the PEGDA/CNTs/LFP and PSH/CNTs/LFP inks. B) Apparent viscosities of various inks as a function of shear rate. C) Storage modulus (G’) and loss modulus (G’’) as a function of shear stress. D) Cross‐linking process of polymer in inks during fiber electrode fabrication. Images of fiber electrodes during E) continuous fabrication, F) bending, G) knotting, H) enwinding, and I) large‐scale production ability.

The polymer and interaction among each component greatly affect the rheological properties of inks, which are critical parameters for fiber electrode fabrication. **Figure** **2**A shows that electrode ink with pure PEGDA exhibited apparent liquid state. In addition, the polymerization process of PEGDA under UV light was drastically prolonged because of the large ratio of CNTs and LFP particles. Thus, the PEGDA/CNTs/LFP ink cannot forming stable fiber architecture which easily to be broken after it was extruded out the needles (Movie S1, Supporting Information), indicating the pure PEGDA cannot be directly used as the polymer component for electrode inks. SA and HEA were added to regulate the rheological properties of the inks, resulting in a stable gel state with higher viscoelasticity. A rheometer was used to evaluate the effects of SA and HEA on ink rheology. Figure 2B shows the apparent viscosities of various inks containing different polymers. The long polymer chain of SA greatly increased the apparent viscosities of inks in the shear stress region. There is also a slight viscosity increase at a low shear rate because of the hydroxyl groups of HEA in the PSH ink. All types of inks exhibited a rapid decrease in apparent viscosity as the shear rate increased because of the orientation and unwinding of the polymer chain and CNTs. This shear‐thinning behavior ensured a fluent extrusion process through the needle.^[^ ^34^ ^]^

Furthermore, the oscillatory amplitude test reveals the influence of SA and HEA on the storage modulus (G’) and loss modulus (G’’). Figure 2C shows that G’ is always higher than G’’ in the lower shear stress region, exhibiting solid‐like behavior. However, the dominant loss modulus in higher stress regions suggests that the inks would transfer to liquid‐like behavior after yield stress.^[^ ^35^ ^]^ For the PEGDA/CNTs/LFP ink, the yield stress is only 0.5 Pa, which is too low to maintain a stable solid‐like state, confirming the liquid statement shown in Figure 2A. In contrast, SA and HEA enhanced the ink modulus and increased the yield stress. The intertwined long polymer chain and abundant hydrogen bonds between the hydroxyl groups in SA and HEA require more shear stress to break, thereby increasing the stability of the solid‐like state of inks. Therefore, with the highest viscosity, modulus, and yield stress, the rheological properties of the PSH/CNTs/LFP ink are suitable for scalable wet spinning of fiber electrodes. It would be fluently extruded under high shear stress in needles and suddenly transfer into a solid‐like state without shear stress, maintaining a stable fiber architecture.^[^ ^36^ ^]^

Figure 2D shows that the prepared PSH/CNTs/LFP ink with suitable rheological properties could continuously establish flexible fiber electrodes because of the physical and chemical crosslinked hybrid polymer networks. After the ink was injected into the coagulation solution, the SA molecules rapidly formed electrostatic interaction with Ca^+^ and established physical crosslinking networks with solvent exchange (from water to ethyl alcohol). The quick SA physical crosslinking ensures the continuous fabrication process of stable fiber electrodes (Movie S2, Supporting Information). So, the time‐consuming polymerization of PEGDA and HEA under UV lighting could be realized subsequently during the whole fabrication process, in order to endow fiber electrodes optimized ionic dynamics.^[^ ^32^ ^]^ Therefore, the hybrid physical and chemical crosslinking networks were achieved in fiber electrodes, regulating the microenvironment of electrodes (include porosity, ionic transportation, and structure stability). Figure 2E–I shows the continuous large‐scale fabrication process and the excellent flexibility of the fiber electrodes. The fiber electrodes could be easily bent, enwound, and even knotted without damage, ensuring the stability of the electrodes during deformation.

### 2.2 Crosslinking Inside the Fiber Electrodes

**Figure 3:**
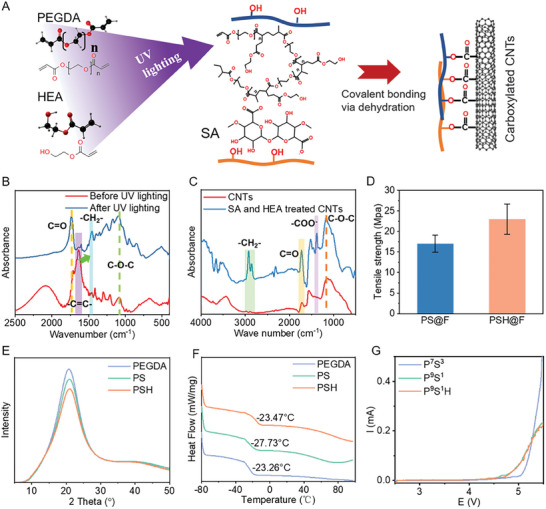
Hybrid cross‐linking network design and property. A) Schematic of chemical bonding in fiber electrodes. B) FT‐IR spectra of the polymer solution before and after UV lighting. C) Bonding between the polymer and carboxylated CNTs via FT‐IR. D) The influence of HEA on the tensile strength of fiber electrode and date are represented as average ± SD (*n* = 3). E) WAXD and F) DSC results of various polymer matrices. G) Electrochemical stability of the spined polymer fiber with various polymer components.

As shown in **Figure** **3**A, PEGDA, SA, HEA, and carboxylated CNTs established hybrid crosslinking networks during the spinning process, which greatly influenced the physical and chemical properties of the polymer matrices and fiber electrodes. The physical cross‐linking networks of SA were first constructed by electrostatic interaction and built fiber architecture during spinning as mentioned above. Based on the basic fiber architecture, HEA and PEGDA would subsequently copolymerize into covalent cross‐linking networks under UV lighting, forming hybrid crosslinking network with SA.^[^ ^32^ ^]^ The SA physical crosslinking networks not only assistant the quick architecture formation but also influence the crosslinking density of PEGDA networks. Additionally, HEA modify the end of the PEGDA molecule with hydroxyl groups, so that the abundant hydroxyl groups of the hybrid polymer network would further chemically bind with carboxylated CNTs through the esterification reaction. The hybrid polymer networks thereby construct a robust electrodes structure, enhancing the mechanical stability of the fiber electrodes.

FT‐IR was used to demonstrate the polymerization and chemical bonding between CNTs. Figure 3B shows the spectra of the polymer mixture before and after UV lighting. The spectra before and after polymerization exhibited characteristic peaks of C═O and C─O─C at 1740 and 1100 cm^−1^, respectively. There is also a strong peak at ≈1640 cm^−1^ corresponding to the amount of ─C═C─ groups in PEGDA and HEA before UV lighting. However, this peak disappeared after UV lighting, and the characteristic peak of ─CH_2_─ rose, indicating that UV lighting induced the additional copolymerization of PEGDA and HEA. Additionally, the FT‐IR spectra in Figure 3C confirm the covalent bonding between polymer networks and carboxylated CNTs. The carboxylated CNTs were mixed with a polymer solution without the photoinitiator (I2959) and subsequently washed. Compared with the spectrum of carboxylated CNTs materials, the characteristic peak of C═O intensified, and additional peaks corresponding to ─CH_2_─ and ─COO^−^ could be observed at ≈2900 and 1380 cm^−1^, respectively. Even after complete washing, the existent groups of HEA and SA molecules on treated CNTs demonstrate that they have been chemically bonded on CNTs. Therefore, compared with PEGDA networks, the PEGDA‐HEA copolymerization networks would possess stronger interaction with CNTs, forming a covalent interaction between the polymer and conductive material skeleton. Because of the reinforced strong interaction via HEA, the mechanical strength of the fiber electrodes enhanced from 17 MPa for PS@CF to 23 MPa for PSH@CF as shown in Figure 3D. The PSH@CF electrodes could withstand more stress originating from deformation and charge/discharge than PS@F, resulting in higher stability.

The introduction of SA and HEA in PEGDA reinforces the bonding networks and regulates the degree of PEGDA crosslinking. A pure PEGDA, PEGDA/SA polymer compound (PS), and PEGDA/SA/HEA polymer compound (PSH) were characterized to further study the effects of SA and HEA. Wide‐angle X‐ray diffraction (WAXD) was used to evaluate the crystallinity of the polymer in Figure 3E,^[^ ^37^ ^]^ which is considerably related to the ionic conductivity of the polymer.^[^ ^38^ ^]^ All the spectra exhibited an intense peak and a week‐wide peak nearby, corresponding to the crystal and amorphous phases of PEGDA, respectively. After normalization of the amorphous peaks, it was easily observed that the intensity of the peak at ≈20° decreased with the addition of SA and HEA, indicating the lower crystallinity of the PEGDA networks. HEA molecules with only one carbon–carbon double bond could modify PEGDA molecules with hydroxyl groups and decrease the cross‐linking density by consuming the polymerization reaction group (C═C). Moreover, the penetrated SA molecules could impede the crystallization of PEGDA via steric hindrance. DSC also tested these polymer matrices to study the glass transition temperature (*T*_g_) of the polymer. As shown in Figure 3F, the PS polymer exhibited a lower *T*_g_ (−27.73 °C) than pure PEGDA (−23.26 °C), which could be attributed to the impeded crystallinity of SA. When modified by HEA, the PSH polymer exhibits a higher *T*_g_ than the PS polymer despite having a lower crystallinity. This could be attributed to the abundant hydrogen bonds inside the molecules generated by the hydroxyl groups in HEA. However, the *T*_g_ of PSH is still lower than that of PEGDA, confirming its lower degree of crystallinity.^[^ ^31^ ^]^ The Li^+^ transport in the polymer is typically recognized as a process generated by the association/disassociation of Li^+^ with polar groups (ether group in PEGDA) and polymer chain movement in amorphous domains. Hence, the PSH polymer matrix possesses favorable ionic conductivity due to the higher chain mobility. Additionally, the porosity of the fiber electrodes would also enhance because of the decrease in cross‐linking density.

Considering that pure PEGDA cannot continuously spin fiber electrodes, the linear sweep voltammetry (LSV) results in Figure 3G exhibited the electrochemical stability of the polymer matrix used in fiber electrodes (P^7^S^3^, P^9^S^1^, and P^9^S^1^H, the superscript represents the mass ratio of PEGDA and SA). As the ratio of SA increased, P^7^S^3^ could withstand higher voltage than P^9^S^1^, indicating the benefit of SA on the electrochemical stability of the polymer. Similar LSV curves of P^9^S^1^ and P^9^S^1^H suggest that adding HEA is harmless to electrochemical stability. These three polymer types are stable under 4 V, which is sufficient for fabricating fiber electrodes with LFP and carboxylated CNTs.

### 2.3 Porous Structure Management for the Fiber Electrodes

**Figure 4:**
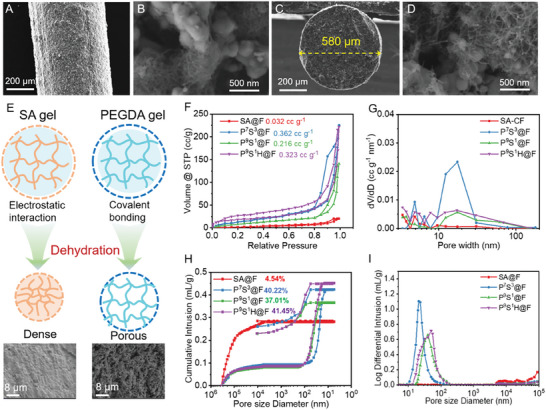
Microstructure and porosity regulation. A,B) Surface and C,D) cross‐section morphology of P^9^S^1^H@F fiber electrodes. E) Schematic of differences in polymer crosslinking networks during dehydration. F) Nitrogen adoption and desorption isotherms and G) pore‐size distribution curves of fiber electrodes with various polymer matrices. H) Mercury intrusion and extrusion curves and their pore‐size distribution curves.

The microscopic images of the prepared fiber electrodes were investigated using scanning electron microscopy, as shown in **Figure** **4**A–D and Figures S1 and S2 (Supporting Information). The fiber electrodes fabricated using the P^9^S^1^H polymer (P^9^S^1^H@F) displayed a uniform dense morphology with a cylindrical structure along its axial direction. The cross‐section of P^9^S^1^H@F exhibited a diameter of 580 µm; thus, the mass loading of the fiber electrodes reached 1.27 mg cm^−1^. The enlarged images in Figure 4B,D shows that the LFP particles were embedded in the polymer matrices and CNTs. As mentioned, the polymer with good ionic conductivity covalently binds with CNTs, constructing robust co‐continued phase ion and electron pathways around LFP particles.^[^ ^39^ ^]^ These strong conductive connections between LFP particles greatly contribute to the excellent stability of fiber electrodes. Moreover, many nanopores exist in fiber electrodes, which are crucial for electrolyte uptake and transfer. The microscopic images of fiber electrodes fabricated with P^9^S^1^ (P^9^S^1^@F) and P^7^S^3^ (P^7^S^3^@F) also exhibited wrapped LFP particles and porous structures (Figures S1 and S2, Supporting Information). However, less covalent bonding between PEGDA networks and CNTs will lead to lower stability. Furthermore, the difference in polymer components results in variations in porosity and ion transport ability.

The hybrid crosslinking network generated the porous structure of fiber electrodes during evaporation. As shown in Figure 4E, the crosslinking network of SA with electrostatic interaction shrinks during the desolvation process, resulting in a density structure,^[^ ^40^ ^]^ while the PEGDA networks with covalent networks would keep the porous structure. This phenomenon highlighted the significant role of PEGDA crosslinking networks in porous structures and was confirmed by the cross‐section microscopic images of pure SA and PEGDA fibers. Therefore, the porosity of the fiber electrodes greatly depends on the cross‐linking density and is regulated by the polymer component and ratio. The BET nitrogen adoption and mercury intrusion porosimeter (MIP), which focus on mesoporous and macropores, respectively, exhibited the porosity and pore‐size distribution of the fiber electrodes to illustrate the effect of polymer components. The MIP and BET results show that the pure SA‐based fiber electrodes (SA@F) have the lowest porosity with an almost flat curve (Figure 4F) and no mercury intrusion at high pressure (after gaps between each fiber were filled) (Figure 4H). These results also confirm that the pure SA polymer cannot offer sufficient pores for electrolyte transfer so that SA@F cannot deliver enough energy storage ability (Figure S3A–C, Supporting Information). The porosity of fiber electrodes was greatly enhanced when PEGDA was utilized as major polymer components. The P^7^S^3^@F possessed a pore volume of 0.362 cm^3^ g^−1^ and porosity of 40.22%, which confirms the importance of PEGDA networks in porous formation. However, with the ratio of SA further decreasing, although P^9^S^1^@F would possess higher ionic dynamics for more PEGDA, its porosity slightly decreased to 37.01%. This might be ascribed to the enhanced crosslinking density of PEGDA networks, which was attributed to the reduction of the steric hindrance effect from SA. The pore distribution in Figure 4G,I also illustrates that the number of pores decreases and shifts to a larger pore‐size range with less shrinking force. Similar with SA, HEA also could decrease the crosslinking density of PEGDA, as shown in Figure 3E. Thus, the P^9^S^1^H@F own higher porosity than P^9^S^1^@F as shown in both BET and MIP results, which would further improve the ionic dynamic.

With the difference on porosity and component ratio, the polymer engineering greatly influence the electrolyte uptake of fiber electrodes (Figure S3D, Supporting Information). Various fiber electrodes were immersion in electrolyte for 10 min. The P^9^S^1^H@F could uptake more electrolyte (101.3 wt.% of dry electrodes) than P^9^S^1^@F because of the higher porosity. Although the P^9^S^1^@F possess lower porosity than P^7^S^3^@F, the P^9^S^1^@F own higher electrolyte uptake than P^7^S^3^@F. The larger pore‐size in P^9^S^1^@F is more suitable for electrolyte to permeate in electrodes. Therefore, the porosity of the fiber electrodes was managed using engineering hybrid polymer networks and influence the electrolyte transfer in electrodes. Combining the preferred ionic conductivity of the polymer and higher porosity, P^9^S^1^H@F would be more favorable for ionic transfer in thick fiber electrodes and offer perfect electrochemical performance.

### 2.4 Ionic Dynamic Property of Thick Fiber Electrodes

**Figure 5:**
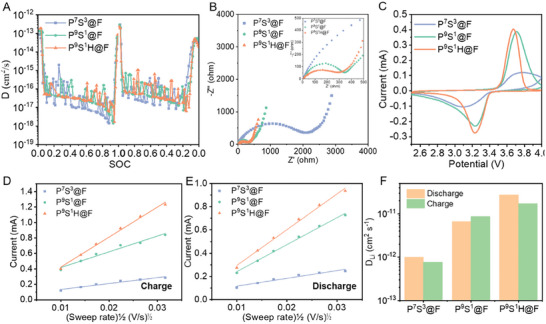
Ionic dynamic optimization in fiber electrodes. A) GITT results of various fiber electrodes during the charge and discharge process. B) Nyquist plots of the fiber electrodes tested at 3.4 V. C) Cyclic voltammetry (CV) curves of the fiber electrodes at a scan rate of 0.1 mV s^−1^. Graphs of normalized peak current versus square root of the scan rate of various electrodes during D) charge and E) discharge process of CV at different scan rates. F) Diffusion coefficient of Li^+^ (D_Li_) obtained from the fitting results of CV at different scan rates.

The impacts of hybrid polymer networking engineering on the ionic dynamic properties of fiber electrodes were investigated, which is important for electrochemical performance of thick fiber electrodes. The galvanostatic intermittent titration technique (GITT) was used to evaluate the ion diffusion process in the solid phase of electrodes.^[^ ^30^ ^]^ The results in **Figure** **5**A exhibited an obvious enhancement in Li^+^ diffusion coefficients (D_Li_) because of the regulation of hybrid polymer networks. The P^7^S^3^@F fiber electrode exhibited the worst ionic dynamic property with the lowest D_Li_. With more PEGDA, although P^9^S^1^@F had lower porosity, it possessed higher D_Li_ in the entire SOC state, revealing the crucial role of the PEGDA polymer chains in ionic transportation in the solid phase. The P^9^S^1^H@F curve almost overlapped that of P^9^S^1^@F during the process, indicating similar D_Li_ in the solid phase. However, adding HEA increased the porosity of P^9^S^1^H@F, thereby exhibiting better ionic dynamic properties for the electrodes.

Cyclic voltammetry (CV) measurements further clarified the effect of polymer components on the redox reaction of LFP and ionic dynamic properties of electrodes. The CV curves obtained at a scan rate of 0.1 mV s^−1^ reflect the redox reaction conditions in these thick fiber electrodes. As shown in Figure 5C, a pair of typical redox peaks of LFP particles could be observed. The current of redox peaks drastically increased, and the polarization decreased when the PEGDA ratio increased from 70% to 90%, which is consistent with the enhanced D_Li_ in the solid phase. Additionally, P^9^S^1^H@F exhibited the highest redox reaction activity and low polarization, demonstrating the benefit of enhanced porosity on Li^+^ transportation compared with P^9^S^1^@F. Furthermore, the apparent D_Li_ of fiber electrodes was investigated by CV tests with various scan rates (Figure S4, Supporting Information) based on the Randles–Sevcik equation (Equation 1).^[^ ^39^ ^]^

(1) $$I_{p}=2.69\times 10^{5}n^{1.5}AD_{Li}^{0.5}C_{0}v^{0.5}$$

where *I*_p_ denotes the peak current (A), ν denotes the scan rate (V s^−1^), n denotes the number of transferred electrons per molecule, which is 1 for LFP, A denotes the surface area of the electrode (cm^2^), and C_0_ denotes the molar concentration of Li^+^ (for LFP, 2.28 × 10^−2^ mol cm^−3^). Consistent with Equation 1, the peak current exhibits a linear relation with the square root of the scanning rate (v^1/2^) for all electrodes (Figure 5D,E). D_Li_ calculated from the fitted slopes are shown in Figure 5F and represent a distinct contribution from polymer engineering.^[^ ^41^ ^]^ Similar to the GITT results, the added PEGDA endows P^9^S^1^@F with a higher average D_Li_ of 7.71 × 10^−12^ cm^2^ s^−1^ than that of P^7^S^3^@F (8.85 × 10^−13^ cm^2^ s^−1^). Moreover, the highest average D_Li_ of 2.25 × 10^−11^ cm^2^ s^−1^ was achieved by P^9^S^1^H@F because of the synthetic effect of enriched pores due to HEA modification and ion transport via PEGDA chains.

Electrochemical impedance spectroscopy (EIS) analysis is another crucial tool for profoundly investigating electronic/ionic transportation behavior variation inside fiber electrodes. These three fiber electrodes were tested to determine the electrode conditions during the charge and discharge processes. The Nyquist plots of all three fiber electrodes in Figure 5B could be divided into a semicircle and an almost straight line with a high sloop, indicating the charge transfer resistance (R_ct_) and Warburg impedance (Z_w_), respectively. According to the fitting results, the R_ct_ values of the P^9^S^1^H@F fiber electrode (241.2 Ω) and P^9^S^1^@F (260.1 Ω) were much lower than P^7^S^3^@F (1580 Ω), indicating a more satisfactory microenvironment of active materials.^[^ ^30^ ^]^ Because of the higher D_Li_ in the solid phase, more Li^+^ reached the surface of the active materials and transferred electrons exhibiting lower resistance. The ionic dynamics of the fiber electrodes were also analyzed using the Warburg impedance parameter, as shown in Equation S1 (Supporting Information).^[^ ^39^ ^]^ The results showed that D_Li_ of the various fiber electrodes were 1.99 × 10^−13^ cm^2^ s^−1^ for P^9^S^1^H@F, 7.50 × 10^−14^ cm^2^ s^−1^ for P^9^S^1^@F, and 2.79 × 10^−14^ cm^2^ s^−1^ for P^7^S^3^@F. These results confirm the effect of polymer engineering on the ionic dynamic properties of thick fiber electrodes, offering a superior microenvironment for LFP particles to realize high linear capacity and excellent electrochemical performance.

### 2.5 Evaluation of the Electrochemical Performance

**Figure 6:**
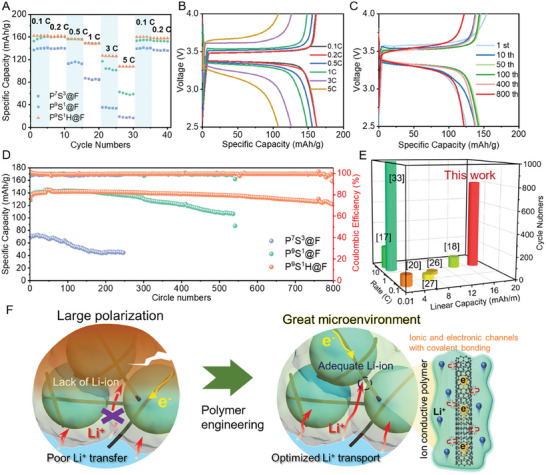
Electrochemical performance of fiber electrodes. A) Performance rate of fiber electrodes with various polymer components. GCD profiles of P^9^S^1^H@F at B) different current densities and C) different cycling numbers at 1 C. D) Long‐term cycling performance of various fiber electrodes at 1 C. E) Electrochemical performance in this study compared with representative fiber electrodes in the literature fabricated using the extrusion method.^[^ ^17^, ^18^, ^20^, ^26^, ^27^, ^33^ ^]^ F) Mechanism of electrochemical performance enhancement.

The electrochemical performance of the thick fiber electrodes was tested using lithium metal as the contact electrodes. The rate performance of various fiber electrodes is shown in **Figure** **6**A, which further demonstrates the enhancement of ionic transfer. Considering the large diameter of the fiber electrodes (≈600 µm), the capacity of the fiber electrodes with a common polymer binder is difficult to deliver for sluggish ionic transfer.^[^ ^42^ ^]^ Even though P^7^S^3^@F had 70% PEGDA, its specific capacity at 0.1 C rate was only 140 mAh g^−1^ and sharply decreased to 17.3 mAh g^−1^ when the rate reached 5 C. In contrast, P^9^S^1^@F yielded a dramatic capacity promotion in the range of 0.1 C–5 C because of the enhanced D_Li_ in P^9^S^1^@F for increased PEGDA content. It could release a specific capacity of 160.8 mAh g^−1^ at a rate of 0.1 C after three cycles, which is close to the theoretical specific capacity of LFP. However, a distinct decline in capacity and severe polarization could still be observed (Figure S5B, Supporting Information) at a rate of 3 C, resulting in low‐capacity retention of 36.6% at 5 C due to the lower porosity. Compared with them, P^9^S^1^H@F achieved an excellent performance rate. As the current density rate increased from 0.1 C to 1 C, the specific capacity of P^9^S^1^H@F was similar to that of P^9^S^1^@F for similar Li^+^ transport ability in the polymer phase, which was confirmed by the GITT results. When the current density rate reached 3 C and 5 C, the specific capacity retained 127 and 108 mAh g^−1^, respectively, indicating an obvious advantage due to the synergistic effect of porosity and ionic conductive polymer regulation.

Figure 6D shows the stability variation of the fiber electrodes through long‐term cycling measurements. These three fiber electrodes were charged and discharged at a rate of 1 C. P^7^S^3^@F exhibited a low initial specific capacity of 69 mAh g^−1^ because of the poor ionic dynamic properties in these thick fiber electrodes (Figure S6A, Supporting Information). Li^+^ barely reaches the active materials at the center of the fiber electrodes, thereby decreasing the capacity delivery.^[^ ^43^ ^]^ In contrast, the optimized Li^+^ ion transport in P^9^S^1^@F and P^9^S^1^H@F resulted in higher initial specific capacities of 128 and 130 mAh g^−1^, respectively (Figure 6C; Figure S6B, Supporting Information). The galvanostatic charge–discharge (GCD) profiles of P^9^S^1^H@F and P^9^S^1^@F also show decreased polarization because of better ionic transfer than that of P^7^S^3^@F.

Additionally, capacity retention reveals the apparent difference in cycling stability. The specific capacity of P^7^S^3^@F quickly decreased to 44.5 mAh g^−1^ after 200 cycles, which remained at only 64.2% of its initial specific capacity. However, the ionic transport improvement endows P^9^S^1^@F with higher specific capacity and better capacity retention because of the lower polarization, which is considerably related to the stress in thick electrodes.^[^ ^44^ ^]^ After the activation process at the beginning of cycling, the specific capacity of P^9^S^1^@F remained stable for 200 cycles but gradually decreased to 109 mAh g^−1^ after 550 cycles (85.2% of initial capacity). In contrast, P^9^S^1^H@F exhibited surprisingly excellent long‐term cycling stability. The specific capacity was maintained at a perfect level during the entire cycling procedure, which declined to only 7.2% of the initial capacity after 800 cycles. The superior stability could be attributed to the cooperation of reduced polarization via ionic dynamic optimization and reinforced bonding between CNTs and polymer networks via HEA modification (Figure 6F). The Coulombic Efficiency date of fiber electrodes during cycling process were also calculated in terms of initial Coulombic Efficiency (ICE) and average Coulombic Efficiency (ACE) in Figure S7A (Supporting Information). The ICE and ACE of P^9^S^1^@F was higher than that of P^7^S^3^@F during cycling, indicating the better reversibility of P^9^S^1^@F. The reversibility further improved for P^9^S^1^H@F (with ACE of 99.37%), which also confirmed the minimum structure degradation and side reaction attributed to the optimized electrochemical environment. Therefore, because of the multiscale microenvironment regulation by polymer engineering, the fiber electrodes displayed an outstanding comprehensive electrochemical performance, including dramatic high mass loading (1.27 mg cm^−1^), linear specific capacity (17.8 mAh m^−1^), and cycling stability, surpassing all the reported extruded fiber electrodes (Figure 6E).^[^ ^17^, ^18^, ^20^, ^26^, ^27^, ^33^ ^]^

**Figure 7:**
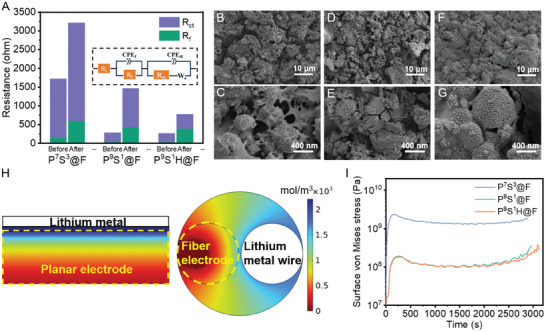
Degradation mechanism of the fiber electrodes and effect of polymer engineering. A) Impedance variation before and after long‐term cycling tests obtained from EIS results. Micromorphology of B,C) P^7^S^3^@F, D,E) P^9^S^1^@F, and F,G) P^9^S^1^H@F. H) Simulated Li‐ion concentration distribution in the planar and fiber electrodes discharged to 3 V with the same thickness (radius). I) Simulated max Von Mises stress at the particle surfaces of LFP in various fiber electrodes during the discharge process.

The EIS results revealed a massive change in the electronic/ionic transfer behavior inside the fiber electrodes after cycling tests. The Nyquist plots (Figure S8A, Supporting Information) after cycling exhibited larger semicircles and lower‐sloop straight lines for P^7^S^3^@F and P^9^S^1^@F but a small semicircle and high‐sloop straight line for P^9^S^1^H@F. The fitting results of EIS in **Figure** **7**A further clarified the differences between these fiber electrodes. The charge transfer resistance, R_ct_, of P^7^S^3^@F increased to a high value of 2616 Ω. The terrible electronic/ionic transfer channels in fiber electrodes resulted in the rapid capacity decline of P^7^S^3^@F. P^9^S^1^@F had a lower R_ct_ than P^7^S^3^@F after cycling, indicating less structural degradation. However, it still suffered structural damage with the R_ct_ of up to 1046 Ω, which was constant with the gradual capacity decrease during cycling. For P^9^S^1^H@F, although the formation of cathode–electrolyte interphase (CEI) enhanced R_f_, as with other electrodes, R_ct_ had much less increment (from 241.2 to 385 Ω). These results indicated that an adequate microenvironment was maintained in P^9^S^1^H@F after long‐term cycling due to polymer engineering.

The microscopic graph of these fiber electrodes after cycling confirms the structural destruction phenomenon. Significant cracks were observed on the surface of P^7^S^3^@F, and significant gaps between the LFP particles were observed (Figure 7B,C). Thus, the electronic transfer inside the fiber was deeply impeded, and numerous LFP particles could even fall off. The cracks and gaps in P^9^S^1^@F were considerably diminished (Figure 7D,E) compared with P^7^S^3^@F, indicating the advantage of PEGDA on the inhibition structure broken. In cooperation with the covalent bonding between PSH networks and CNTs due to HEA, the surface of P^9^S^1^H@F remained almost intact. The polymer matrix still wrapped the active materials and CNTs, preserving favorable electronic and ionic co‐continued channels (Figure 7F,G). Thus, P^9^S^1^H@F exhibited high‐capacity retention with a well‐maintained microenvironment, even after 800 cycles.

Furthermore, the COMSOL simulation further reveals the reason for the severe structural damage in fiber electrodes with high mass loading and the influence of ionic dynamic optimization on cycling stability. The stability of thick electrodes was deeply impacted by polarization, which aggravated the stress generated by the swelling of active material particles.^[^ ^42^, ^44^ ^]^ Most reported fiber electrodes usually exhibit lower cycling life than planar electrodes with the same active materials. These could be attributed to the deteriorated polarization problem because of the unique 1D architecture and would be more severe in thick fiber electrodes with high mass loading. Figure 7H shows that Li^+^ distribution in the planar electrode is more uniform than that in fiber electrodes with the same parameters (porosity, ionic diffusion coefficients, conductivity, and active material ratio). The ionic concentration at the inner area of the fiber electrodes is much lower than that at the surface, and the higher polarization in the fiber electrodes induces the high stress generated by active materials inside the electrode.^[^ ^44^ ^]^ Figure S8B (Supporting Information) shows the maximum surface Von Mises stress of the LFP particles in the electrodes calculated during the discharge process, which confirms the higher surface Von Mises stress in the fiber electrodes.

Thus, thick fiber electrodes have to face a higher challenge of structural stability during cycling. Due to polymer engineering, the ionic dynamics were optimized for porosity and solid D_Li_ to reduce the polarization and stress in thick fiber electrodes. As shown in Figure 7I, the surface Von Mises stress in the fiber electrodes with the P^9^S^1^H@F and P^9^S^1^@F parameters greatly decreased compared with that of P^7^S^3^@F. The lower stress during cycling endows P^9^S^1^@F and P^9^S^1^H@F with less structural damage and a longer cycling life. Moreover, with the extra covalent bonding between the polymer and CNTs, the reinforced mechanical property further enhances the structural stability during cycling; thus, P^9^S^1^H@F exhibited a superior long‐term cycling life.

**Figure 8:**
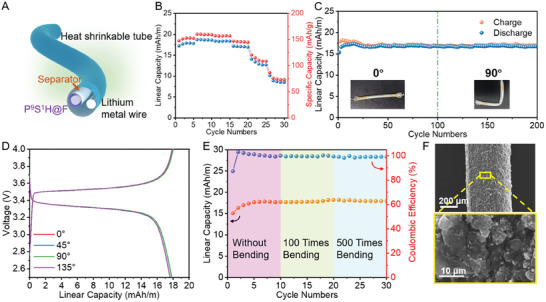
Flexibility of fiber‐shaped batteries. A) Schematic of assembled fiber‐shaped batteries. B) Performance rate of fiber‐shaped batteries. C) Cycling performance of fiber‐shaped batteries with or without bending. D) GCD profiles of assembled fiber‐shaped batteries with various bending angles. E) Capacity retention of fiber‐shaped batteries after various bending times. F) Microstructure of the fiber electrodes after 500 bending cycles.

With superior electrochemical performance, P^9^S^1^H@F was paired with a lithium metal wire to assemble a fiber‐shaped battery (FLB) (**Figure** **8**A), which exhibited the flexibility of the fiber electrodes. The assembled FLB achieved a capacity of 18.6 mAh m^−1^ and 160 mAh g^−1^ at 0.2 C, as shown in Figure 8B. The linear capacity gradually decreased to 17.1, 12.7, and 8.6 mAh m^−1^ at 1 C, 3 C, and 5 C, respectively. Additionally, FLB with a high linear capacity of 17 mAh m^−1^ exhibited high‐capacity retention after 200 cycles, whether bending at 90° or not (Figure 8C). Figure 8D shows that the charge and discharge curves of FLB at various bending angles from 0° to 135° almost overlap. No distinct capacity degradation of FLB was observed, and the microstructure of P^9^S^1^H@F maintained integrity with only wrinkles generated by folding, even after 500 bending cycles to 90°. No serious self‐discharge phenomenon was observed during 2 weeks, indicating the integrity and stability of FLB as shown in Figure S9 (Supporting Information). Above all, FLB demonstrated excellent flexibility and deformation durability, showing its application potential as a flexible energy‐storage solution with high linear energy density.

## 3 Conclusion

In this study, we proposed a polymer engineering strategy to endow thick fiber electrodes with a favorable inner microenvironment, including high ionic dynamics and reinforced covalent bonding. The fiber electrodes prepared using facial ink‐extrusion technology exhibit high active material mass loading (1.27 mg cm^−1^) and impressive linear capacity (17.8 mAh m^−1^). PEGDA crosslinking networks adjusted using HEA and SA construct porous fiber structures and enhance Li^+^ transportation through polymer chains, thereby optimizing ionic dynamics. HEA further establishes covalent bonding with carboxylated CNTs, which reinforces the stability of the electronic/ionic transfer channels. Moreover, the crucial role of ionic dynamics in stress reduction inside thick fiber electrodes during cycling was demonstrated. The fiber electrodes achieved long‐term cycling (92.8%, 1 C, 800 cycles) because of reduced stress via ionic dynamics optimization and rich covalent bonding, even under ultrahigh mass loading. This study offers a new perspective for achieving high‐energy‐density fiber batteries with excellent electrochemical performance and appeals for more attention to special polymer design in fiber electrodes, which might be valuable for developing wearable energy‐storage fabric.

## 4 Experimental Section

See Supporting Information for details.

## Conflict of Interest

The authors declare no conflict of interest.

## Supporting information

Supporting Information

Supplemental Movie 1

Supplemental Movie 2

## Data Availability

The data that support the findings of this study are available from the corresponding author upon reasonable request.
