# Supplementary material for: Polymer Engineering Enables High Linear Capacity Fiber Electrodes by Microenvironment Regulation
Source: Adv Sci (Weinh). 2024 Apr 26;11(28):2309461. doi: 10.1002/advs.202309461 (PMC11267365; doi:10.1002/advs.202309461)
Supplement: Supplementary file 1 — Supporting Information [file ADVS-11-2309461-s003.pdf]

ADVANCED SCIENCE
Open Access

Supporting Information

for *Adv. Sci.*, DOI 10.1002/advs.202309461

Polymer Engineering Enables High Linear Capacity Fiber Electrodes by Microenvironment Regulation

Yuan Li, Yibo Wang, Yan Liu, Fang Yan, Zhenwei Zhu, Xibang Chen, Jingyi Qiu, Hao Zhang and Gaoping Cao**

Supporting Information

Polymer engineering enables high linear capacity fiber electrodes by microenvironment regulation

Yuan Li, Yibo Wang, Yan Liu, Fang Yan, Zhenwei Zhu, Xibang Chen, Jingyi Qiu, Hao Zhang, Gaoping Cao**

Materials and methods

Materials:

$CaCl_2$ powders and $LiFePO_4$ particles with an average diameter of 400 nm were purchased from Shanghai Macklin Biochemical Co., Ltd. PEGDA (1000 MW), SA (10000 MW), HEA, and carboxylated CNTs were provided by Shanghai Aladdin Biochemical Technology Co., Ltd. The electrolyte, 1.0 mol/L lithium hexafluorophosphate ($LiPF_6$) in EC/DEC (1:1 v/v), was provided by Guangdong Card New Energy Technology Co., Ltd.

Preparation of inks used for extrusion

The electrode inks with the tolerant rheological property were prepared with the polymer solution, carbonylated CNTs, and LFP to represent common active materials. The SA, PEGDA, and HEA were mixed in deionized water as the designed ratio with magnetic stirring for over eight hours, named PSH polymer solution. Then, carboxylated CNTs and LFP with the same mass were added into the solution with sufficient mechanical stirring. The polymer solution without HEA was also prepared (PS) and ratio of SA was changed to research the function of each components. The mass ratio of PEGDA and SA was noted at the superscript (P^7S^3, P^9S^1, and P^9S^1H).

The fabrication of thick fiber electrodes

The prepared inks were injected into the coagulation solution through a nozzle with an inner diameter of 0.8mm by a microinject pump (LANDE LD P2020). The coagulation solution containing equal mass deionized water and ethyl alcohol with 1.5 wt.% $CaCl_2$. During the spinning process, the gel fiber in coagulation was exposed under the 365nm UV lighting with an intensity of 441 mW/cm^2. After the solvent exchange process, the fiber electrodes were washed with pure ethyl alcohol and further dry under UV lighting. The prepared fiber electrodes with P^xS^yH polymer, carboxylated CNTs, and LFP were named P^xS^yH@F (P^7S^3@F, P^9S^1@F, and P^9S^1H@F). The collected fiber electrodes were used for battery assembling and further characterization.

Characterization

The rheological of the electrode inks were analyzed using a shear-controlled rheometer (Anton Paar MCR 302). FR-IR spectra were obtained by an Infrared Spectrometer (Thermo Scientific Nicolet iS5). The tensile strength of the fiber electrodes were measured by YG006 single fiber strength meter. The crystallinity and crosslinking density of the polymer were evaluated by Homelab Wide-angle X-ray diffractometer and Differential Scanning Calorimeter Q2000. Scanning

electron microscopy (SEM) at FEG650 at 10 kV examined the surface morphology and structure. Nitrogen adsorption-desorption isotherms were determined in surface area and pore size analyzers (Micromeritics ASAP 2460). Mercury porosimeter (Micromeritics AutoPore V 9620) was used further to evaluate the porosity and distribution in fiber electrodes.

Electrochemical characterization:

The electrochemical performance of fiber electrodes was evaluated using CR 2032-type coin cells to avoid the influence of encapsulation, assembled with fiber electrodes (1.5 cm) as work electrode, lithium metal as the reference electrode and a double-layer Celgard membrane as the separator. Lithium hexafluorophosphate ($LiPF_6$, 1 mol/L) in EC/DEC (1:1 v/v) was used as the electrolyte. Electrochemical impedance spectra were obtained in the frequency range from 0.1 Hz to 1000 kHz at 3.4V with an electrochemical workstation (Solartron SI 1260 and 1287). The cells were charge to 3.4 V via linear sweep voltammetry (0.1 mV/s) and immediately change to EIS test by software program, obtaining the electronic/ionic transportation behaviour during the discharge process. Cyclic voltammetry curves of fiber electrodes were tested by an electrochemical workstation (Princeton Applied Research PARSTAT) from 2.5 V to 4 V with different scan rate. The cells' GITT, cycling performance, and rate performance are measured by the Land CT2001A battery test system at room temperature with a voltage range from 2.5 V to 4 V. As for fiber-shaped batteries, the prepared fiber electrode was tied on the Al tab and wrapped by a Celgard membrane. Lithium metal wire was paired with the fiber electrode and packaged by a heat shrink tube. The electrolyte was injected into the tube.

Supplemental Figures

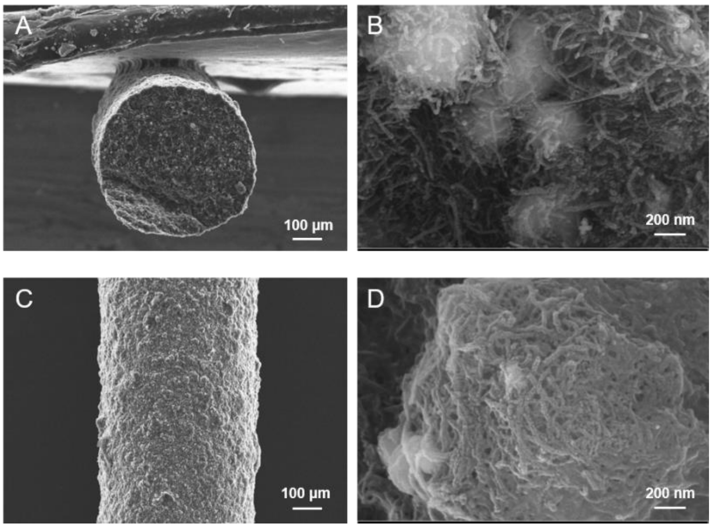

Figure S1. The microscopic graph of (A-B) cross-section and (C-D) surface of P^7S^3@F.

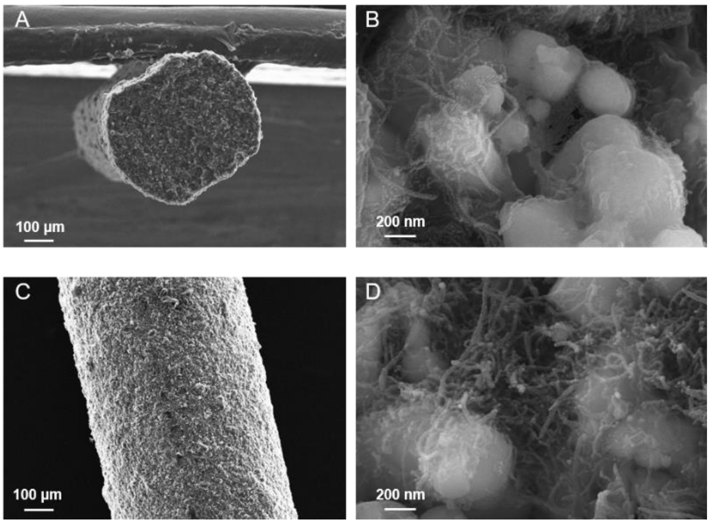

Figure S2. The microscopic graph of (A-B) cross-section and (C-D) surface of the P[9]S[1]@F.

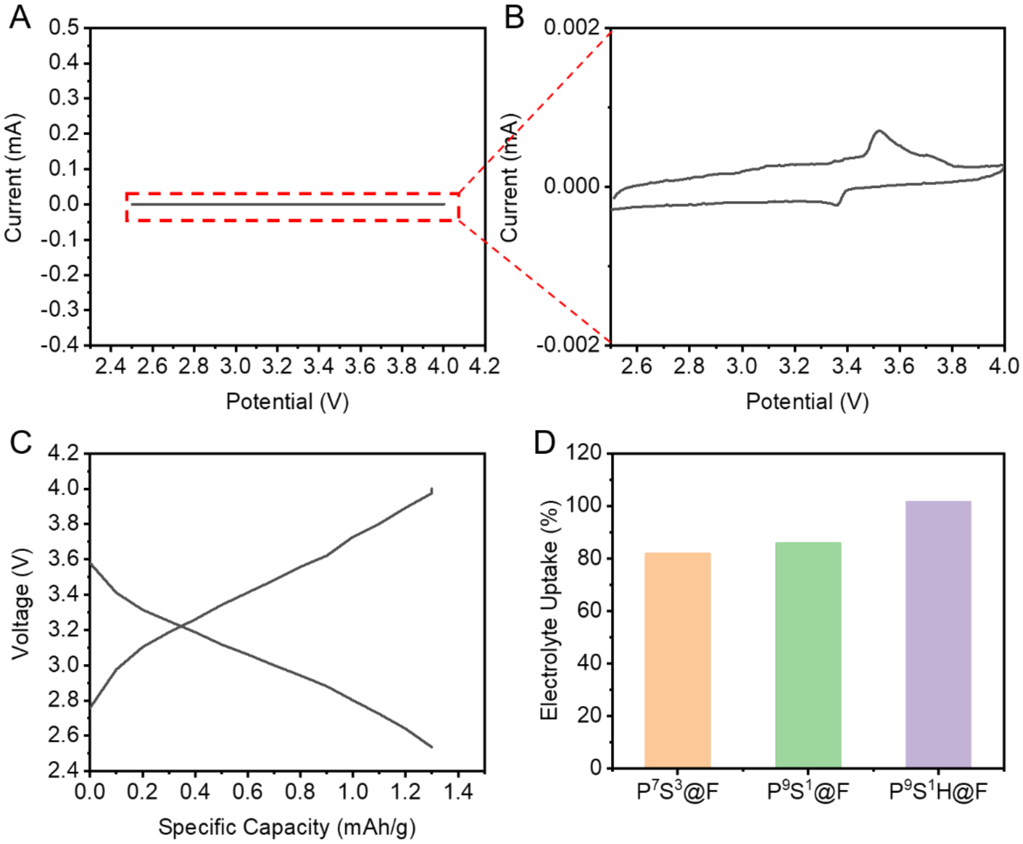

Figure S3. (A) the CV curve of SA@F and (B) enlarged view. (C) The charge and discharge curves of SA@F at 0.1 C. (D) The electrolyte uptake of various fiber electrodes after immersing in electrolyte for 10 min.

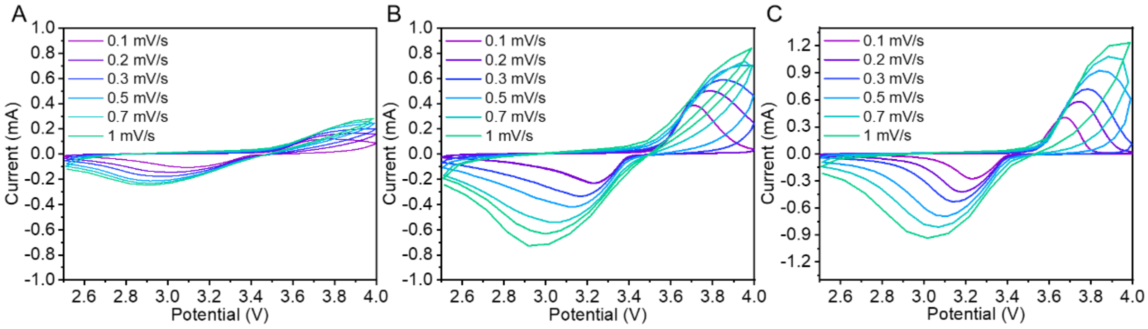

Figure S4. The CV curves of the (A) P^7S^3@F, (B) P^9S^1@F, and (C) P^9S^1H@F with various scan rates.

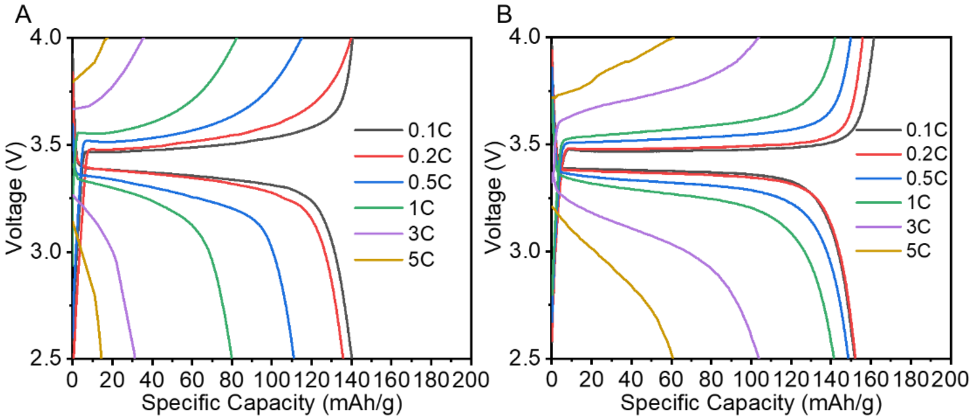

Figure S5. The GCD profiles of (A) P^7S^3@F and (B) P^9S^1@F at different rates.

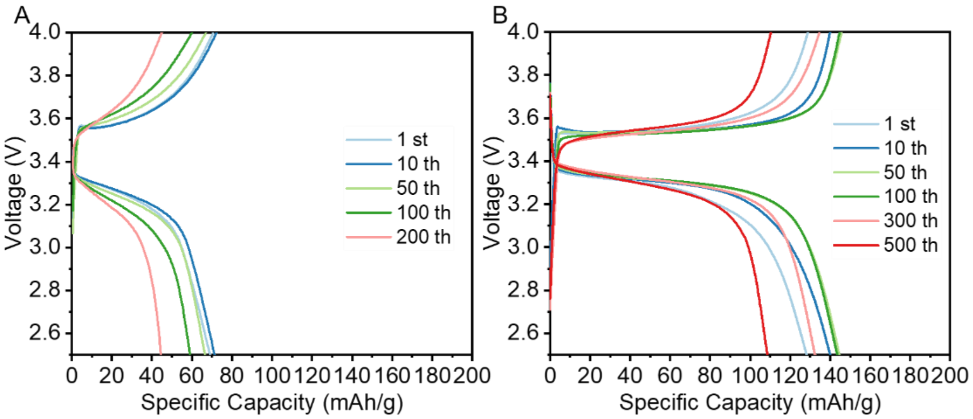

Figure S6. The GCD profiles of (A) P^7S^3@F and (B) P^9S^1@F after different cycling numbers.

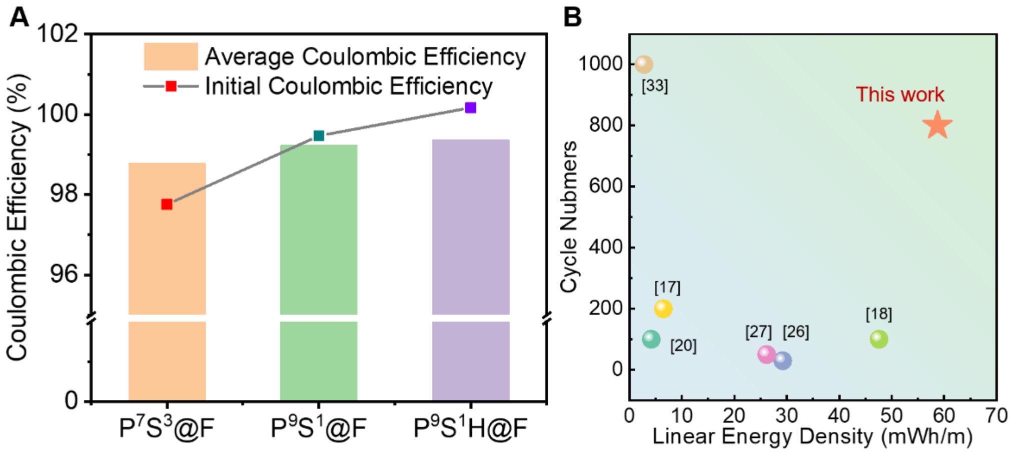

Figure S7. (A) The initial Coulombic Efficiency (ICE) and average Coulombic Efficiency (ACE) of fiber electrodes during long-term cycling tests. (B) The linear energy density and cycle numbers of fiber electrodes compared with other representative fiber electrodes in the literature.

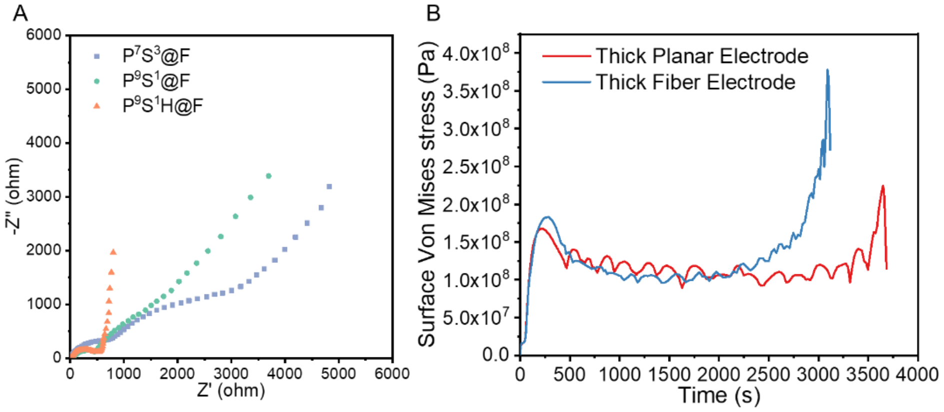

Figure S8. (A) The Nyquist plots of fiber electrodes at 3.4V after cycling tests. (B) The calculated max surface Von Mises stress according to COMSOL simulation in thick planar electrode and fiber electrode during discharging to 3V.

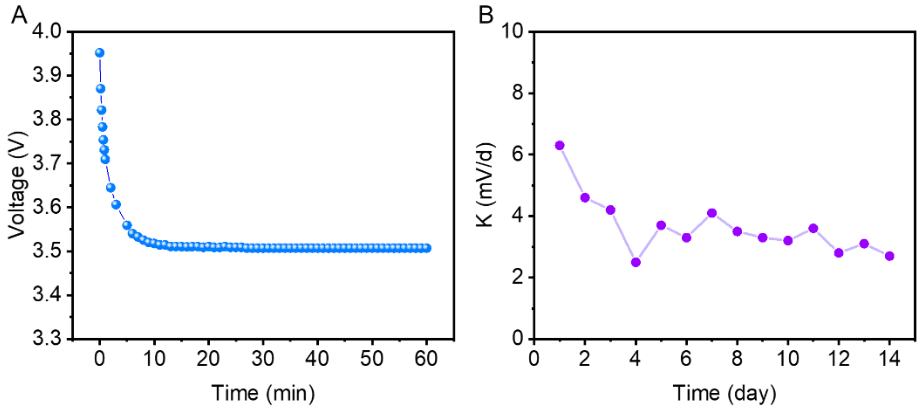

Figure S9. (A) The Open Circle Voltage (OCV) of the FLB after charge to 4 V (B) The variation rate of the OCV (K = ΔOCV/t) of FLB during the subsequent 14 days.

Equation S1. The Li-ion diffusion coefficients (D_{Li}) were calculated from EIS results according to the following equation:

$$D_{Li} = \frac{R^2T^2}{2A^2n^4F^4C_0^2\sigma^2}$$

Where R is the gas constant, T is the absolute temperature (298 K), A is the surface area of the fiber electrodes (cm^2), n is the number of electrons transferred per molecule during charge and discharge reaction (for LFP, n=1), F is the Faraday constant, C_0 is the molar concentration of Li^+ in LFP, and σ is the Warburg factor obtained from Z' in Warburg diffusion region of Nyquist plots.
